# Morphological and molecular pathological features of the breast carcinoma with choriocarcinomatous features: A case report and a literature review

**DOI:** 10.3389/fonc.2023.984425

**Published:** 2023-03-08

**Authors:** Jingchun Xu, Yi Xu, Cheng Xu, Cong Wang

**Affiliations:** Department of Pathology, The First Affiliated Hospital of Nanjing Medical University, Nanjing, China

**Keywords:** breast, carcinoma, choriocarcinoma, human chorionic gonadotropin, pathological features, breast carcinoma with choriocarcinomatous features

## Abstract

Here we present a rare case of breast cancer with both invasive ductal carcinoma and choriocarcinoma components in a 55-year-old woman. Firstly, the serum human chorionic gonadotropin level showed 15.9mIU/ml preoperatively. And adequate immunohistochemical tests were performed on the specimen. Secondly, High-throughput sequencing was performed to detect the molecular characteristics of the two components, respectively. Then, DNA short tandem repeat (STR) analysis confirmed the homology of the two components, indicating the somatic origin of choriocarcinoma components. Finally, the clinical course and pathological characteristics of the case were reviewed and a literature search for other cases was performed.

## 1 Introduction

The presence of trophoblastic differentiation or nongestational choriocarcinoma in carcinoma is rare, but it is described in various organs including the breast. Carcinomas that exhibit trophoblastic differentiation often show aggressive behaviour (1–3). Breast carcinoma with choriocarcinomatous features (BCCF) is a rare variant of breast carcinoma and was first reported by Saigo and Rosen in 1981 (4). To date, a total of 19 cases of BCCF have been reported (4–16). Here, we reported the morphological and molecular pathological features of a 55-year-old woman with BCCF and review the previous literature about BCCF.

## 2 Case presentation

A 55-year-old woman with regular menstruation, presented with a four-year history of a left breast lump, and no previous history of hydatidiform moles or choriocarcinoma has been reported. Furthermore, the patient underwent a modified radical mastectomy. Histopathologically, the tumor revealed breast infiltrating ductal carcinoma (BIDC) with areas of choriocarcinomatous features, giant cells and intense atypia, and a sentinel lymph node macrometastasis. In addition, the Serum human chorionic gonadotropin (HCG) level showed 15.9mIU/ml in the preoperative time. The patient underwent chemotherapy after surgery and the postoperative serum HCG level was <5mIU/ml.

**Figure 1:**
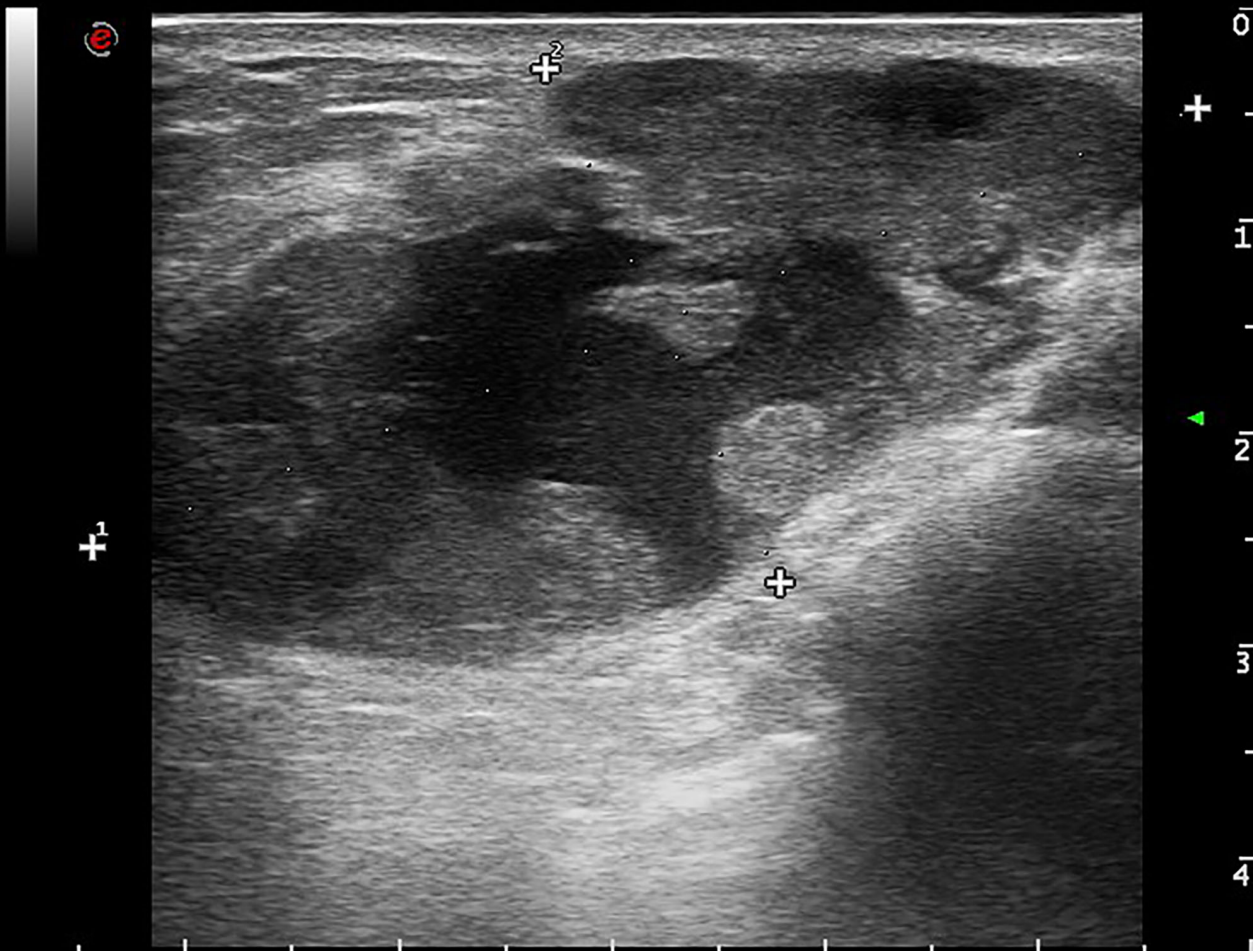
Breast imaging. Ultrasonography shows cystic solid compound echo with a size of 56*44*27mm of the left breast.

Mammography showed a dense mass with a small calcification lesion on the left breast, 30*27mm in size. The breast ultrasound confirmed the presence of cystic solid compound echo, with a size of 56*44*27mm (**Figure 1**).

Grossly, the tumor was 40*32*20 mm, the cut surface was gray-red and gray-yellow, and some areas were cystic solid. Necrosis, hemorrhage, and cystic degeneration are seen in some areas.

**Figure 2:**
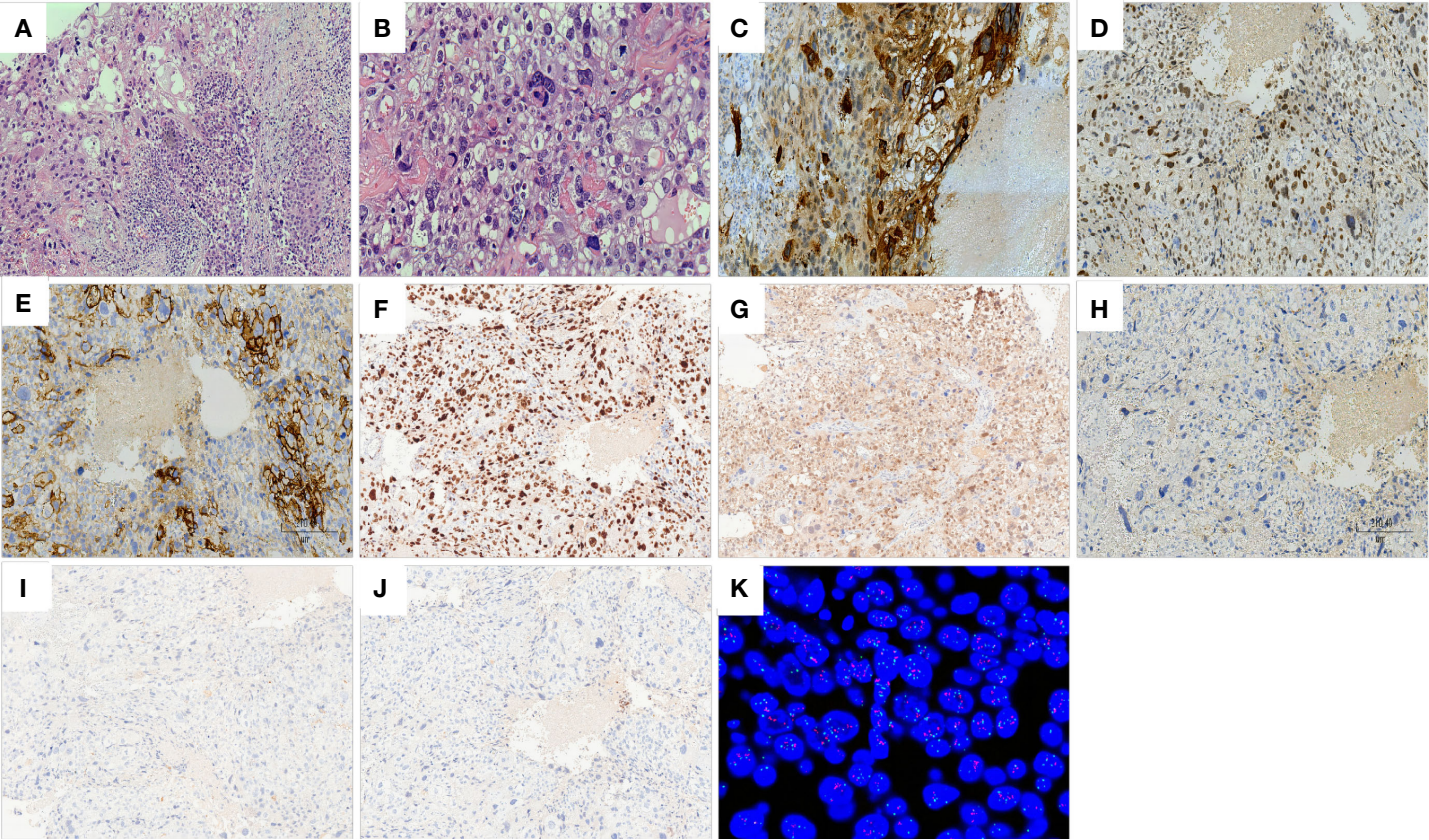
Histology of breast carcinoma with choriocarcinomatous features (BCCF). Hematoxylin–eosin staining (×100): close transition from the BIDC to the choriocarcinomatous areas **(A)**. Hematoxylin–eosin staining (×200): giant cells with prominent pleomorphic nuclei and abundant eosinophilic and vacuolated cytoplasm resembling cytotrophoblastic and syncytiotrophoblastic cells **(B)**. Immunohistochemistry of choriocarcinoma components (×100): HCG **(C)**, GATA3 **(D)**, HER2 **(E)**, KI67 **(F)**, P53 **(G)** are positive; ER **(H)**, PR **(I)**, AR **(J)** are negative. Fluorescence *in situ* hybridization (FISH): HER-2 gene amplification (HER2/CEP17 ratio = 2.31) **(K)**.

Microscopically, tumor composition is complex, and morphology is diverse. About 75% were choriocarcinomatous differentiated components, giant cells with prominent polymorphic nuclei and abundant eosinophilic and vacuolated cytoplasm were observed in the context of massive hemorrhagic necrosis, similar to cytotrophoblastic and syncytiotrophoblastic cells. Focal cystic structures are also seen, with choriocarcinomatous cells lining the cyst wall. About 25% had an invasive breast carcinoma component, with nested sheets of tumor cells and high-grade Nottingham histology (mitotic count score, 2; nuclear pleomorphism score, 3; glandular lumen formation score, 3). In addition to the two morphological features described above, the invasive breast cancer area has various forms. Some of the mucin-producing areas (intracellular and extracellular mucin) can be seen, and signet-ring-like cells can be seen focally. However, no clear intraductal carcinoma components are found and atypical apocrine proliferations can be seen in the surrounding individual ducts. Furthermore, there was a close transition from the BIDC to the choriocarcinomatous areas on several slides (**Figure 2**). One of the sentinel nodes is a macrometastase, histologically presenting as BIDC.

Immunohistochemistry (IHC)was performed on automated Benchmark ULTRA platform (Ventana Medical Systems Inc.) with standard protocols using commercially available antibodies. BIDC components revealed positive reactivity to ER (90% 3+), PR (50% 2+), AR (90% 2+), HER2 (2+), Ki67 (40%), P53 (10%), GATA3. Choriocarcinoma components showed that HCG, HER2 (2+), Ki67 (80%), P53 (20%), and GATA3 were positive and ER, PR, and AR were negative. Pathologist scored the HER2 IHC using the 2018 ASCO/CAP guidelines.

HER2 FISH was performed on paraffinized pre-treatment biopsy tissue samples using HER2 DNA dual probe kit (Abbott Laboratories, Abbott Park, IL) according to the manufacturer^’^s instructions. According to the 2018 ASCO/CAP guidelines, the HER2 FISH results is considered Positive: HER2 gene amplification (HER2/CEP17 ratio = 2.31) (**Figure 2**). Next-generation sequencing (NGS) was performed on formalin-fixed-paraffin-embedded (FFPE) tumour tissue blocks. FoundationOne CDx examined replacement, insertion, and deletion changes, copy number changes, and specific gene rearrangements in 324 genes, along with genome signatures including tumor mutation burden (TMB) and microsatellite instability (MSI). Mutations in MSH6, PIK3CA, TP53, RAD51, FOXL2, CHEK1, PIK3R1, RAD21, RARA, and ERBB2 genes were found in the BIDC region. Whereas, mutations in MSH6, PIK3CA, TP53, RAD51, FOXL2, CHEK1, PIK3R1, ERBB2, SPOP, and CIC genes were found in the trophoblastic neoplasm region. The overlap of the two components accounted for 60% of the mutant genes and the mutation pattern and locus was consistent. The TMB of both components is 1Mut/Mb and the MSI status is stable.

**Figure 3:**
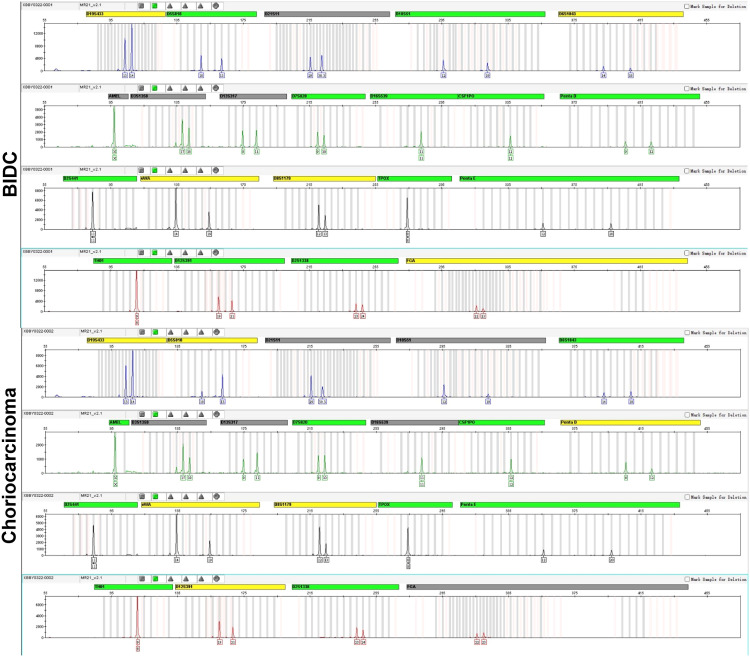
DNA STR analysis. DNA STR analysis reveals the genomic DNA alleles of the choriocarcinomatous differentiated components are identical to those of the BIDC components, exhibiting that the choriocarcinoma is nongestational.

An appropriate amount of the test material was extracted using the Microread Genomic DNA Kit, 20 STR loci and sex identification loci were amplified using the MicroreaderTM21 ID System, and the PCR products were detected using the ABI 3730xl genetic analyzer. The test results were analyzed using GeneMapperID-X software (Applied Biosystems). To investigate the origin of choriocarcinoma, we performed DNA short tandem repeat (STR) analysis of BIDC and choriocarcinoma components respectively. The results of the STR analysis are shown in **Figure 3**. Genomic DNA alleles of the choriocarcinomatous differentiated components are identical to those of the BIDC components, confirming that the choriocarcinoma was nongestational.

In this case, the combination of surgery and chemotherapy was used in treating BCCF. After 12 months of follow-up, the patient is still alive, and no tumor metastasis or recurrence was found.

## 3 Discussion

Trophoblastic differentiation or nongestational choriocarcinoma occurring in association with a somatic carcinoma has been described at multiple sites including the ovary, breast, colon, and urinary tract (1–3). This is a rare event, the pathogenesis of which is not fully clear. The present study describes a case of BCCF in a 55-year-old woman. BCCF is a distinguishing variant of breast cancer. The most common histopathological subtype of the carcinomatous component is BIDC (1–3), as seen in the present case. Mucoid carcinoma of the breast has also been documented (5). Histologically, this part of the tumor has typical choriocarcinoma morphology and immunohistochemical characteristics, with tubulocystic glandular spaces lined by a predominance of giant cells showing prominent pleomorphic nuclei, abundant eosinophilic and vacuolated cytoplasm resembling cytotrophoblastic and syncytiotrophoblastic cells (4, 8). The tumor cells show positive immunoreactivity to HCG, as well as an elevated serum HCG (4). In this study, we found that the choriocarcinoma region was focally positive for HCG. **Table 1** summarizes the clinicopathological features of 18 previously reported cases of BCCF, in addition to the present case. The median age of the patients was 51.5 years (range, 31-71 years). Most tumors were in the right breast and all cases were positive for HCG antibodies in IHC studies (4–16).

**Table 1:** Summary of reports in literature.

Case	Year	Age (years)	Localization	Size (cm)	LN	M	Treatment	Follow-up	ICD-DCIS	HCG	HPL	CK	HER2	ER	PR	P53	Reference
1	1981	55	L	2.5	–	Lung	Operation	Died	+	+	/	/	/	/	/	/	(4)
2	1990	71	R	2.5	20/21	Lymph nodes	Mastectomy, ETX	DFS	Mucoid	+	/	/	/	/	/	/	(5)
3	1999	38	R	1	44/44	Multiple organ	Operation	Died	+	+	/	/	–	–	–	/	(6)
4	2001	50	R	7	0/20	–	Operation	/	+	+	–	+	–	+	–	/	(7)
5	2002	48	R	2.5	0/16	–	Mastectomy, CTX, RTX	DFS	+	+	+	+	+	–	–	+	(8)
6		49	R	1.6	–	/	Mastectomy	Lost	+	+	+	+	–	–	–	–	
7		58	R	4	12/19	–	Mastectomy, CTX, RTX	DFS	+	+	+	+	–	+	–	+	
8		59	R	2.5	4/19	/	Mastectomy, CTX, RTX	Lost	–	+	+	+	+	–	–	–	
9	2004	38	R	5	/	Multiple organ	Operation	Died	–	+	/	+	/	–	–	/	(9)
10		54	R	10	–	Multiple organ	Neo CTX, MRM, CTX, RTX	Died	+	+	/	+	–	–	–	/	
11	2006	56	R	3.5	–	–	MRM	DFS	+	+	/	+	/	–	+	/	(11)
12	2008	50	R	4	0/19	–	Operation	DFS	+	+	/	+	–	–	–	+	(10)
13		53	L	3.5	0/10	–	Operation	DFS	+	+	/	/	+	–	–	–	
14	2010	31	L	/	/	/	Neo CTX, MRM	/	+	+	/	+	–	–	–	/	(12)
15	2011	41	R	3	/	Multiple organ	CTX	Died	–	+	/	/	/	/	/	/	(13)
16	2013	56	R	3.5	–	–	MRM	DFS	+	+	/	+	/	–	+	/	(14)
17	2014	32	L	3.2	–	Lung,kidney	Mastectomy, CTX	DFS	–	+	/	+	–	–	–	/	(15)
18	2022	49	L	8.5	–	Lymph nodes,Lung	Neo CTX, MRM	DFS	+	+	/	/	/	–	–	/	(16)
19	Present	55	L	4	1/23	–	Mastectomy, CTX	DFS	+	+	+	+	+	–	–	+	

+, positive; -, negative;/, unknown; L, center; R, right; LN, lymph node status; M, metastasis status; MRM, modified radical mastectomy; DFS, disease-free survival; CTX, chemotherapy; RTX, radiotherapy; ETX, endorine therapy; Neo CTX, neoadjuvant chemotherapy.

The mechanism of development of a mixed tumor including a component of choriocarcinoma is unclear. It could be the result of breast carcinoma trophoblast differentiation, or it could represent 2 independent primary tumors where trophoblastic tumors originate in pregnancy. Furthermore, three hypotheses have been postulated: 1. dedifferentiation of epithelial cells into choriocarcinomas (17, 18). 2. germ cells that fail to complete their migration to the gonads (18) and 3. multidirectional tumor differentiation from a common stem cell (19). To delineate their clonal relationship, by using microscopic segmentation, we analyzed the components of BIDC and choriocarcinoma by NGS and DNA STR. The common mutations were MSH6, PIK3CA, TP53, RAD51, PIK3R1, ERBB2, FOXL2, and CHEK1. Among the common mutations, MSH6, PIK3CA, TP53, RAD51, PIK3R1, and ERBB2 are common mutations in BIDC with the same mutation pattern, and their TMB and MSI are the same, suggesting that the two components are of the same clonal origin. DNA STR analysis revealed the origin of the choriocarcinoma as nongestational, as the genotype of BIDC components entirely corresponded with that of choriocarcinomatous differentiated components.

HER2 is a member of the epidermal growth factor receptor (EGFR) family, and its products are transmembrane signaling molecules with close structural homology, and both are involved in cell transformation and tumor pathogenesis (20). Previous reports have found that HER2 expression was significantly greater in complete mole and choriocarcinoma than in partial mole and normal placenta, and that gestational trophoblastic disease with HER2 amplification and expression in combination with DNA hyperploidy showed higher proliferation and more aggressive behavior (21). HER2 amplification was found in both BIDC and choriocarcinoma components by immunohistochemistry, FISH, and NGS. Anti-HER2 targeted therapy may be effective for both tumor components. BCCF is a highly malignant tumor of the breast and shows aggressive behavior in most cases with many patients presenting with lymph nodes and distant metastasis (4–16). Current therapeutic strategies for BCCF mainly consist of endocrine therapy, surgery, and chemotherapy (6, 8, 22). The chemotherapy in the BCCF regimen remains unclear (9, 12, 22). Surgery is generally considered to be effective in treating BCCF. In our case, the combination of surgery and chemotherapy was used in treating BCCF, the patient is still alive.

The pathological feature of BCCF is similar to that of choriocarcinoma in the female genital tract (22). BCCF must be distinguished from metastatic choriocarcinoma of the breast. History of previous gestational trophoblastic disease (molar pregnancy or choriocarcinoma) will support the diagnosis of metastasis, while the demonstration of *in situ* carcinoma or small foci of typical invasive ductal carcinoma will support the diagnosis of primary breast carcinoma. Many studies have shown that STR analysis can distinguish gestational from nongestational neoplasms and can provide useful information about the conceptual types of etiology. In our case, STR analysis revealed the BIDC components’ genotype entirely matched that of the choriocarcinoma components, confirming the nongestational type. In addition, it should be differentiated from breast neoplasms with multinucleated giant cells (MGCs). MGCs on breasts should suggest many diagnostic possibilities. These giant cells could be osteoclastic, metaplastic, or sarcomatoid in origin (23, 24). Immunochemical stains play a significant role in concluding the histogenesis of a tumor and the origin of MGCs. The presence of prominent cytologic atypia and positive staining for HCG support the differentiation of choriocarcinoma and the malignant nature of these cells in our case.

Combined with previous reports and this case, BCCF is characterized by high invasion and high metastasis rate. Therefore, identifying the components of choriocarcinoma is crucial for establishing appropriate treatment strategies. Immunohistochemistry and even molecular genetics are often needed to assist in diagnosis. Finally, as the pathogenesis of the disease is not clear, it suggests that the pathogenesis and treatment plan of the disease should be further explored and studied.

## Data availability statement

The original contributions presented in the study are included in the article/supplementary material. Further inquiries can be directed to the corresponding author.

## Ethics statement

Written informed consent was obtained from the individual(s) for the publication of any potentially identifiable images or data included in this article.

## Author contributions

JX: writing and editing of the manuscript and review of final submission. YX: collection of data and figure preparation. YX and CX were involved in histopathological diagnosis. CW: collection of data, editing of the manuscript, figure preparation, and review of final submission. All authors contributed to the article and approved the submitted version.
